# Prohibitin 3 gives birth to a new lateral root primordium

**DOI:** 10.1093/jxb/erac175

**Published:** 2022-06-24

**Authors:** Le Luo, Yuanming Xie, Wei Xuan

**Affiliations:** MOA Key Laboratory of Plant Nutrition and Fertilization in Lower-Middle Reaches of the Yangtze River and State Key Laboratory of Crop Genetics and Germplasm Enhancement, Nanjing Agricultural University, Nanjing 210095, China; MOA Key Laboratory of Plant Nutrition and Fertilization in Lower-Middle Reaches of the Yangtze River and State Key Laboratory of Crop Genetics and Germplasm Enhancement, Nanjing Agricultural University, Nanjing 210095, China; MOA Key Laboratory of Plant Nutrition and Fertilization in Lower-Middle Reaches of the Yangtze River and State Key Laboratory of Crop Genetics and Germplasm Enhancement, Nanjing Agricultural University, Nanjing 210095, China

**Keywords:** Auxin, founder cell, lateral root, nitric oxide, PHB3

## Abstract

This article comments on:

**Li S, Li Q, Tian X, Mu L, Ji M, Wang X, Li N, Liu F, Shu J, Crawford NM, Wang Y.** 2022. PHB3 regulates lateral root primordia formation via NO-mediated degradation of AUXIN/INDOLE-3-ACETIC ACID proteins. Journal of Experimental Botany **73,**4034–4045.

**Plant lateral roots (LRs) initiate when a small group of pericycle cells are primed to undergo cell division to form LR primordia (LRPs). This process involves a complex gene regulatory network. In Arabidopsis, an auxin-dependent AUX/IAA14/28–ARF7/19–GATA23/LBD16 signaling cascade is known to control the LR initiation. However, it is largely unknown how auxin signaling is regulated. In this issue,** Li *et al.* (2022 **) identified prohibitin 3 (PHB3) as a regulator of LR initiation in Arabidopsis. PHB3 affects the accumulation of endogenous nitric oxide (NO), which leads to the degradation of IAA14 and IAA28, thereby inducing the expression of GATA23 and LBD16 to activate LR initiation.**

LR founder cells are a small set of xylem pole pericycle (XPP) cells that maintain high auxin signaling but have not divided yet. At the longitudinal axis, the LR founder cell is restricted to only one single cell or two adjacent XPP cells (Torres-Martinez *et al.*, 2020). Once LR founder cells perceive high auxin input, they undergo nuclear migration and anticlinal asymmetric cell division to generate a stage I LRP (a single pericycle cell layer with a central core of small cells) (De Smet *et al.*, 2008; De Rybel *et al.*, 2010). This earliest LR event involves founder cell specification and divisions to establish a single layer structure, and is defined as LR initiation (De Smet, 2012), which determines the birth of a new LRP.

LR initiation is controlled by the plant hormone auxin. The core auxin signaling components auxin/indole-3-acetic acid (AUX/IAA) proteins IAA14/SOLITARY-ROOT (Okushima *et al.*, 2007), IAA18/POTENT (Perianez-Rodriguez *et al.*, 2021), and IAA28, interact with AUXIN RESPONSE FACTOR7 (ARF7) and ARF19, to regulate the founder cell specification and LR initiation. Downstream of auxin signaling, MEMBRANE-ASSOCIATED KINASE REGULATOR4 (MAKR4) (Xuan *et al.*, 2015), and the transcription factors (TFs) GATA23 (De Rybel *et al.*, 2010) and LATERAL ORGAN BOUNDARIES-DOMAIN 16/ASYMMETRIC LEAVES2-LIKE 18 (LBD16/ASL18) (Goh *et al.*, 2012), are induced by auxin to promote LR initiation. However, it was unknown whether auxin signaling is modulated during LR initiation.

In this issue, Li *et al.* (2022) identified prohibitin 3 (PHB3) as a new regulator of LR initiation. PHB has previously been known as a tumor suppressor in mammals, and is also involved in plant stress responses (Wang *et al.*, 2020). NO may also act as a signal molecule to promote LR formation (Correa-Aragunde *et al.*, 2006; Schlicht *et al.*, 2013), though the mechanism remains unclear. In this study, the authors revealed that PHB3 causes accumulation of endogenous NO, which leads to the degradation of IAA14 and IAA28 and increased expression of *GATA23* and *LBD16*, ultimately modulating the founder cell specification and asymmetric division.

## PHB3 affects the accumulation of nitric oxide to activate LR initiation

The authors first focused on the regulation by PHB3 of LRP development. LR density was significantly lower in *phb3* mutants due to the reduced LRP number and density. Histological analysis showed that *PHB3* was strongly expressed in stage I of LRPs. These results suggest that PHB3 is required for LR initiation. Gravistimulation assays showed that PHB3 may also regulate LRP development. The new LRPs induced by 18 h gravistimulation were predominantly inhibited or delayed at stage I in the *phb3* mutants while LRPs in the wild type were mainly at the second, third, and fourth stages.

A previous study has raised the possibility that NO and auxin are involved in the inhibition of LRP formation in *phb3* mutants (Wang *et al.*, 2010). Exogenous IAA or SNAP (*S*-nitroso-*N*-acetylpenicillamine; an NO donor) treatments significantly induced the expression of *GATA23* and *LBD16*, and affected LR density, which indicated that IAA and NO promote LR initiation. Interestingly, treatments with 1-naphthylphthalamic acid (NPA; an auxin transport inhibitor), cPTIO (an NO scavenger), and their combination caused a similar inhibitory effect on LR formation, indicating that NO and IAA might function in the same pathway to regulate LR formation. Application of IAA did not rescue LR density in the cPTIO-treated seedlings, indicating that NO is required for the auxin-induced LR development. The fluorescence of the NO indicator DAF-FM DA showed that NO was mainly distributed around LRPs and induced by IAA in the wild type while in *phb3* mutants the induction was much lower. By RNA-seq analysis, differentially expressed genes in the wild type and *phb3* were clustered in three Gene Ontology (GO) terms: root development; auxin biosynthesis and response; and NO response. Therefore, PHB3 is involved in regulating NO- and auxin-induced LR formation.

## PHB3 regulates the degradation of IAA proteins through endogenous NO accumulation

RNA-seq and GO analysis revealed that the expression of LR-related genes and auxin metabolic and biosynthetic pathways were altered by NO treatment, strongly indicating that the NO regulation of LR formation may involve auxin signaling pathways. Notably, almost all the genes up-regulated by NO treatment were down-regulated in the *phb3* mutant, and vice versa. These observations suggest that PHB3 and NO activate the same signaling components for LR formation. NO was previously reported to regulate root development by affecting the activity of the auxin receptor TIR1 and PIN1-dependent polar auxin transport (Fernandez-Marcos *et al.*, 2011; Terrile *et al.*, 2012). To further analyze the mechanism by which NO regulates auxin-induced LR development, the authors focused on four auxin-responsive genes (*GATA23*, *ARF19*, *LBD16*, and *LBD18*). Only *GATA23* and *LBD16* were induced by both IAA and SNAP, and exogenous SNAP could strongly induce the LR density in the wild type, while induction was much weaker in the mutants of *GATA23* and *LBD16*. Thus, GATA23 and LBD16 may contribute to NO-induced LR formation. The expression of *GATA23* and *LBD16* was significantly reduced in *phb3* mutants, and ectopic expression of *GATA23* and *LBD16* could rescue the mutant phenotype of *phb3*. These results confirmed that GATA23 and LBD16 work downstream of PHB3 and NO to modulate LRP formation.

The expression of *GATA23* and *LBD16* is negatively regulated by IAA28 and IAA14. The expression of *IAA28* and *IAA14* was also changed in *phb3* mutants, which indicated the PHB3 modulation of IAA28 and IAA14 transcripts. Protein degradation assays revealed that IAA28–green fluorescent protein (GFP) and IAA14–GFP signals disappeared within 5 min of IAA treatment in wild-type roots, while the signals persisted in *phb3* roots even after 10 min of IAA treatment, and the degradation could be restored by NO supply. These results indicate that NO functions in the degradation of IAA28 and IAA14 and that PHB3 regulates LRP initiation by modulating NO-mediated AUX/IAA degradation.

## Perspectives

These findings reveal a novel ‘PHB3–NO’ signaling module regulating LR initiation through modulation of the canonical AUX/IAA-mediated auxin signaling cascade (Box 1). Remarkably, the cyclic degradation and accumulation of IAA proteins create oscillating signals in the oscillation zone, which in turn triggers pre-branch site formation (Kircher and Schopfer, 2018; Xuan *et al.*, 2020). This process occurs even preceding LR initiation. Considering the strong regulation by ‘PHB3–NO’ on AUX/IAA protein degradation, it will be interesting to probe the role of PHB and NO signals in oscillation signals and periodic pre-branch site formation. Furthermore, LR development is influenced by environmental signals (Lavenus *et al.*, 2013; Motte *et al.*, 2019; Duan *et al.*, 2021). PHB3 and NO both interact with reactive oxygen species to mediate plant development responses to environmental stresses (Scheler *et al.*, 2013; Kong *et al.*, 2018), thus it raises the question of whether the PHB3–NO signal module may serve as an intermediator that adapts LR development patterns to the ever-changing environmental conditions.

**Box 1. The molecular network of PHB3 and NO in regulating LR initiation**

Prohibitin 3 (PHB3) induces nitric oxide (NO) accumulation to promote lateral root (LR) initiation. During LR initiation, a pair of xylem pole pericycle cells are primed by auxin signaling and specified as founder cells that undergo asymmetric cell division to develop as a stage I LR primordium (LRP). This process is activated by an auxin/indole-3-acetic acid (AUX/IAA)–AUXIN RESPONSE FACTOR (ARF)-dependent auxin signaling cascade. PHB3 accumulates NO in pericycle cells and LRPs, and NO in turn triggers the degradation of AUX/IAA28 and IAA14 and the activation of ARFs, thereby inducing the expression of transcription factor genes *GATA23* and *LATERAL ORGAN BOUNDARIES-DOMAIN 16* (*LBD16*) to promote LR initiation and LRP development.

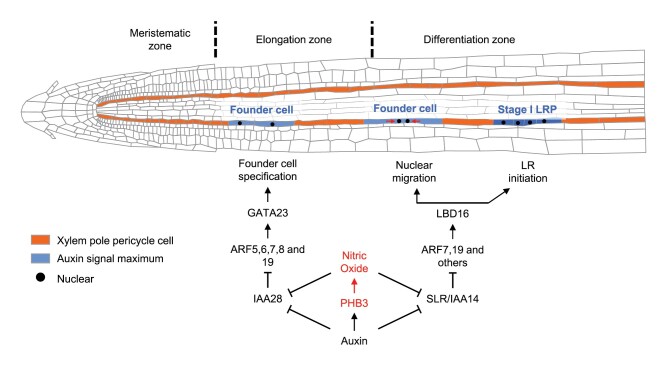

## References

[CIT0001] Correa-Aragunde N , Graziano M, Chevalier C, Lamattina L. 2006. Nitric oxide modulates the expression of cell cycle regulatory genes during lateral root formation in tomato. Journal of Experimental Botany 57, 581–588. 16410257 10.1093/jxb/erj045

[CIT0002] De Rybel B , Vassileva V, Parizot B, et al. 2010. A novel aux/IAA28 signaling cascade activates GATA23-dependent specification of lateral root founder cell identity. Current Biology 20, 1697–1706. 20888232 10.1016/j.cub.2010.09.007

[CIT0003] De Smet I. 2012. Lateral root initiation: one step at a time. New Phytologist 193, 867–873. 22403823 10.1111/j.1469-8137.2011.03996.x

[CIT0004] De Smet I , Vassileva V, De Rybel B, et al. 2008. Receptor-like kinase ACR4 restricts formative cell divisions in the Arabidopsis root. Science 322, 594–597. 18948541 10.1126/science.1160158

[CIT0005] Duan X , Xu S, Xie Y, et al. 2021. Periodic root branching is influenced by ight through an HY1–HY5–auxin pathway. Current Biology 31, 3834–3847.e5 e3835. 34283998 10.1016/j.cub.2021.06.055

[CIT0006] Fernandez-Marcos M , Sanz L, Lewis DR, Muday GK, Lorenzo O. 2011. Nitric oxide causes root apical meristem defects and growth inhibition while reducing PIN-FORMED 1 (PIN1)-dependent acropetal auxin transport. Proceedings of the National Academy of Sciences USA 108, 18506–18511. 10.1073/pnas.1108644108 PMC3215072 22021439

[CIT0007] Goh T , Joi S, Mimura T, Fukaki H. 2012. The establishment of asymmetry in Arabidopsis lateral root founder cells is regulated by LBD16/ASL18 and related LBD/ASL proteins. Development 139, 883–893. 22278921 10.1242/dev.071928

[CIT0008] Kircher S , Schopfer P. 2018. The plant hormone auxin beats the time for oscillating light-regulated lateral root induction. Development 145, dev169839. 30389851 10.1242/dev.169839

[CIT0009] Kong X , Tian H, Yu Q, et al. 2018. PHB3 maintains root stem cell niche identity through ROS-responsive AP2/ERF transcription factors in Arabidopsis. Cell Reports 22, 1350–1363. 29386120 10.1016/j.celrep.2017.12.105

[CIT0010] Lavenus J , Goh T, Roberts I, Guyomarc’h S, Lucas M, De Smet I, Fukaki H, Beeckman T, Bennett M, Laplaze L. 2013. Lateral root development in Arabidopsis: fifty shades of auxin. Trends in Plant Science 18, 450–458. 23701908 10.1016/j.tplants.2013.04.006

[CIT0011] Li S , Li Q, Tian X, Mu L, Ji M, Wang X, Li N, Liu F, Shu J, Crawford NM, Wang Y. 2022. PHB3 regulates lateral root primordia formation via NO-mediated degradation of AUXIN/INDOLE-3-ACETIC ACID proteins ACID proteins. Journal of Experimental Botany 73, 4034–4045. 10.1093/jxb/erac115 35303089

[CIT0012] Motte H , Vanneste S, Beeckman T. 2019. Molecular and environmental regulation of root development. Annual Review of Plant Biology 70, 465–488. 10.1146/annurev-arplant-050718-100423 30822115

[CIT0013] Okushima Y , Fukaki H, Onoda M, Theologis A, Tasaka M. 2007. ARF7 and ARF19 regulate lateral root formation via direct activation of LBD/ASL genes in Arabidopsis. The Plant Cell 19, 118–130. 17259263 10.1105/tpc.106.047761 PMC1820965

[CIT0014] Perianez-Rodriguez J , Rodriguez M, Marconi M, et al. 2021. An auxin-regulable oscillatory circuit drives the root clock in Arabidopsis. Science Advances 7, eabd4722. 33523850 10.1126/sciadv.abd4722 PMC7775764

[CIT0015] Scheler C , Durner J, Astier J. 2013. Nitric oxide and reactive oxygen species in plant biotic interactions. Current Opinion in Plant Biology 16, 534–539. 23880111 10.1016/j.pbi.2013.06.020

[CIT0016] Schlicht M , Ludwig-Muller J, Burbach C, Volkmann D, Baluska F. 2013. Indole-3-butyric acid induces lateral root formation via peroxisome-derived indole-3-acetic acid and nitric oxide. New Phytologist 200, 473–482. 23795714 10.1111/nph.12377

[CIT0017] Terrile MC , Paris R, Calderon-Villalobos LI, Iglesias MJ, Lamattina L, Estelle M, Casalongue CA. 2012. Nitric oxide influences auxin signaling through S-nitrosylation of the Arabidopsis TRANSPORT INHIBITOR RESPONSE 1 auxin receptor. The Plant Journal 70, 492–500. 22171938 10.1111/j.1365-313X.2011.04885.x PMC3324642

[CIT0018] Torres-Martinez HH , Hernandez-Herrera P, Corkidi G, Dubrovsky JG. 2020. From one cell to many: morphogenetic field of lateral root founder cells in *Arabidopsis thaliana* is built by gradual recruitment. Proceedings of the National Academy of Sciences USA 117, 20943–20949. 10.1073/pnas.2006387117 PMC7456154 32817465

[CIT0019] Wang Y , Ries A, Wu K, Yang A, Crawford NM. 2010. The Arabidopsis prohibitin gene PHB3 functions in nitric oxide-mediated responses and in hydrogen peroxide-induced nitric oxide accumulation. The Plant Cell 22, 249–259. 20068191 10.1105/tpc.109.072066 PMC2828708

[CIT0020] Wang D , Tabti R, Elderwish S, Abou-Hamdan H, Djehal A, Yu P, Yurugi H, Rajalingam K, Nebigil CG, Desaubry L. 2020. Prohibitin ligands: a growing armamentarium to tackle cancers, osteoporosis, inflammatory, cardiac and neurological diseases. Cellular and Molecular Life Sciences 77, 3525–3546. 32062751 10.1007/s00018-020-03475-1 PMC11104971

[CIT0021] Xuan W , Audenaert D, Parizot B, et al. 2015. Root cap-derived auxin pre-patterns the longitudinal axis of the Arabidopsis root. Current Biology 25, 1381–1388. 25959963 10.1016/j.cub.2015.03.046

[CIT0022] Xuan W , De Gernier H, Beeckman T. 2020. The dynamic nature and regulation of the root clock. Development 147, dev181446. 32014866 10.1242/dev.181446

